# High Frequency Jet Ventilation in Respiratory Failure Secondary to Respiratory Syncytial Virus Infection: A Case Series

**DOI:** 10.3389/fped.2016.00092

**Published:** 2016-08-30

**Authors:** Kevin M. Valentine, Ajit A. Sarnaik, Hitesh S. Sandhu, Ashok P. Sarnaik

**Affiliations:** ^1^Section of Critical Care, Department of Pediatrics, Riley Hospital for Children, Indiana University, Indianapolis, IN, USA; ^2^Department of Pediatrics, Critical Care Division, Children’s Hospital of Michigan, Wayne State University, Detroit, MI, USA; ^3^Department of Pediatrics, Critical Care Division, Le Bonheur Children’s Hospital, University of Tennessee, Memphis, TN, USA

**Keywords:** respiratory insufficiency, respiratory acidosis, respiratory syncytial virus, high frequency ventilation, high frequency jet ventilation, bronchiolitis

## Abstract

**Objective:**

To describe the utility of high frequency jet ventilation (HFJV) as a rescue therapy in patients with respiratory failure secondary to respiratory syncytial virus (RSV) that was refractory to conventional mechanical ventilation (CMV).

**Design:**

Descriptive study by retrospective review.

**Setting:**

Pediatric intensive care unit at a tertiary care children’s hospital.

**Patients:**

Infants on mechanical ventilation for respiratory failure due to RSV.

**Interventions:**

Use of HFJV.

**Main Results:**

Eleven patients were placed on HFJV. There was sustained improvement in ventilation on HFJV with a mean decrease in PCO_2_ of 9 mmHg at 24 h and 11 mmHg at 72 h. There were no significant changes in oxygenation by oxygenation index. No patients required extracorporeal support or suffered pneumothorax, pneumomediastinum, or subcutaneous emphysema. Ten out of 11 (91%) patients survived to discharge from the hospital.

**Conclusion:**

High frequency jet ventilation may represent an alternative therapy for RSV-induced respiratory failure that is refractory to CMV.

## Introduction

Respiratory syncytial virus (RSV) is one of the most common causes of lower respiratory tract infection in children worldwide, causing a substantial disease burden. In a large population-based study in the USA, RSV bronchiolitis was responsible for 22–24% of outpatient visits and an estimated 75,000–125,000 annual hospital admissions in the USA (1). Of hospitalized children with RSV-related illness, approximately 10–20% require critical care due to acute respiratory insufficiency and require admission to the pediatric intensive care unit (PICU) with intubation and mechanical ventilation (2). About 5% of intubated patients require more support than conventional ventilation can provide.

Ventilation index (VI), which takes into account PaCO_2_ in relation to respiratory support, has been correlated with an increased risk of death in children with acute respiratory failure, and it has been proposed that alternative modalities be sought when VI is greater than 65 (3). In these patients, the use of rescue therapies such as high frequency oscillatory ventilation (HFOV) and extracorporeal membranous oxygenation (ECMO) has been advocated (4–6). Patient characteristics associated with more severe disease and adverse outcomes include prematurity, young age, chromosomal abnormalities, cardiac disease, and neuromuscular disease (1, 5, 6).

Infants with respiratory failure from RSV can present with diverse clinical presentations characterized by distinct pathophysiology. Some patients primarily exhibit increased lower airway resistance with airway edema, mucus plugging, and dynamic hyperinflation. Other patients primarily exhibit a disease of decreased static compliance similar to acute respiratory distress syndrome (ARDS), with alveolar disease, decreased functional residual capacity, and intrapulmonary shunting (7, 8). Infants who develop ARDS from RSV are more likely to be younger and have premorbid conditions such as prematurity, congenital heart disease, and immunodeficiency compared to those who have more obstructive disease (7).

High frequency jet ventilation (HFJV) has been described in patients with air leak/bronchopleural fistula (9), in infants with pulmonary interstitial emphysema (10), and ARDS (11, 12). We report our single institution experience with HFJV in RSV respiratory failure with severe derangements in oxygenation and ventilation not responding to conventional mechanical ventilation (CMV).

## Materials and Methods

A list of intubated children with RSV who were treated with HFJV from October 2010 through May 2012 was obtained from the Virtual PICU database (Virtual PICU Systems, LLC). In these patients with clinical bronchiolitis, RSV was confirmed by rapid antigen test or PCR from respiratory tract secretions.

Medical records were retrospectively examined for the following data: age of patient, premorbid conditions, sex, weight, days of illness, indication for intubation, peak conventional ventilator settings, arterial or capillary blood gases before and after the initiation of HFJV, and HFJV settings. Total time spent on each mode of mechanical ventilation was calculated and any additional or rescue therapies to improve ventilation, such as airway lavage with or without bronchoscopy and the use of heliox were documented. Full conventional mechanical ventilator support was defined as tidal volume of 7–9 mL/kg with minimum set rate of 26 breaths per minute. The VI was calculated as [respiratory rate × (PIP-PEEP) × PCO_2_]/1000.

This study was approved by the Institutional Review Board at Wayne State University School of Medicine and Detroit Medical Center.

### Inclusion/Exclusion Criteria

Patients were included if they had RSV bronchiolitis and transitioned to HFJV from CMV. Reasons for the patients to be transitioned from CMV to HFJV were categorized as: (a) hypercapnic respiratory failure if patients had a PIP greater than 35 cmH2O for more than 6 h associated with persistent respiratory acidosis (pH ≤ 7.25, PCO_2_ > 60 mmHg) or (b) hypoxemic respiratory failure with ARDS as defined by Berlin criteria (13).

### Ventilation Strategy

All intubated RSV patients were managed with pressure-controlled, synchronized intermittent mandatory ventilation (SIMV) as the initial ventilation mode. Puritan Bennett 840 (Covidien, Mansfield, MA, USA), Hamilton G-5 (Reno, NV, USA) and E vent–Inspiration 5i (San Clemente, CA, USA) were used for mechanical ventilation.

High frequency jet ventilation was delivered by Life Pulse HFJV (Bunnell Inc., Salt Lake City, UT, USA) in tandem with a conventional ventilator. HFJV settings were chosen with an initial FiO_2_ of 1.0, and a rate of 360–420 per minute with an inspiratory time of 0.02 sec. Peak inflation pressure was initiated at 40 cmH_2_O and titrated based on PCO_2_ clearance. The PEEP of the conventional ventilator functions as the source for the mean airway pressure (MAP) of the system and was titrated to achieve a MAP 2–3 cmH_2_O higher than the MAP on conventional ventilation prior to initiating HFJV. A beginning rate of five conventional ventilator breaths per minute was titrated down to zero as MAP was optimized (see http://www.bunl.com/technical.html for diagram of HFJV setup).

### Statistical Analysis

For descriptive statistics, categorical variables are presented using absolute counts and percentage. Continuous variables are presented using medians and range. Bivariate correlations were performed with continuous data and Spearman’s correlation with ordinal data. Comparisons conducted between variables were done with related-samples Wilcoxon Signed Rank test. Adverse event data are reported using raw numbers, percentages, and ratios. SPSS version 21.0 was utilized for analyses; *p* < 0.05 was considered to be significant.

## Results

During the time frame of evaluation, there were 75 patients admitted with RSV-related respiratory failure. Eleven patients met the inclusion criteria for the study; all had community acquired RSV. One patient received 2 courses of HFJV, thus constituting a total of 12 courses. Baseline characteristics and brief clinical summaries of the patients are presented in Table 1. Nine out of the 11 patients were prematurely born, and 3 were on oxygen at baseline. One patient had trisomy 21 with an unrepaired ventricular septal defect. Only one subject was a previously healthy, full-term infant. Two patients initially met criteria for mild ARDS while the other nine met criteria for moderate to severe ARDS based on the 2012 Berlin definition (13).

**Table 1:** **Patient characteristics and clinical course summary**.

Pt	Age (wks)	Wt (kg)	Comorbid	VI^a^	OI^a^	HFJV days	MV days	ICU days	Other clinical events
1	26	2.4	24-week premature female, home oxygen	132	32	7	19	27	Bronchoscopies (4), surfactant, no complications, survived
2	2	1.7	35-week premature male, chronic lung disease	54	20	7	24	33	iNO, bronchoscopies (2), no complications, survived
3	3	4.7	Previously healthy female	71	7	4	7	8	No complications, survived
4	156	14.2	26-week premature male	^b^	12	7	9	14	PCO_2_ at the time of HFJV initiation 92 mmHg. No complications, survived
5.1	13	4.2	34-week premature male, home oxygen for first 6 weeks of life	52	11	1	23	32	1st HFJV course: HFJV 1 day discontinued for hypercarbia, received heliox and surfactant. 2nd HFJV course: HFJV 5 days, bronchoscopies (3). Subglottic stenosis, survived
5.2				85	8	5	23	32	
6	8	4.8	36-week premature male	52	11	1	5	6	No complications, survived
7	4	3.0	34-week premature male	62	12	6	13	16	No complications, survived
8	8	4.4	34-week premature female, liver abscess	^b^	37	9	24	30	PCO_2_ at HFJV initiation 71 mmHg. Inotropes, bronchoscopies (2), no complications, survived
9	4	2.9	Trisomy 21, VSD, 35-week premature female	33	14	10	17	37	Bronchoscopy once, no complications, survived
10	3	4	31-week premature female, on home oxygen	40	17	6	11	13	Bronchoscopies (2), no complications, survived
11	26	4.7	24-week premature female, on MV first 4 months of life, on home oxygen	^b^	28	2	10	12	Pre-HFJV on HFOV for ARDS, pneumothorax, surfactant. HFJV 2 days with worsening ARDS. On inotropes, multiple organ failure, died

*^a^VI and OI calculated at time of HFJV initiation where data available*.

*^b^Conventional mechanical ventilation data not available to calculate VI at baseline for patients 4, 8, and 11*.

For all 12 courses, HFJV was used as a rescue therapy for ventilatory failure with hypoxia and/or hypercapnia refractory to CMV. The pre-HFJV VI was available for nine patients. The mean and median VI were 65 and 54, respectively (range of 33–132). All subjects had hypercapnic respiratory failure defined as an elevated PCO_2_ with respiratory acidosis despite full conventional mechanical support. Other therapies used in the clinical care of these patients are presented in Table 2. Of note, no patient received preventative RSV immune globulin despite the eligibility of all but one patient.

**Table 2:** **Therapies (*n* = 12 HFJV courses); data are absolute counts (%)**.

Inhaled nitric oxide	2 (17)
Heliox	1 (8)
Mucolytic therapy	3 (25)
Surfactant	3 (25)
Palivizumab (Synagis^R^)	0 (0)
Systemic corticosteroids	0 (0)
ECMO	0 (0)
Antibiotics	6 (50)
Bronchoalveolar lavage	7 (58)

**Figure 1:**
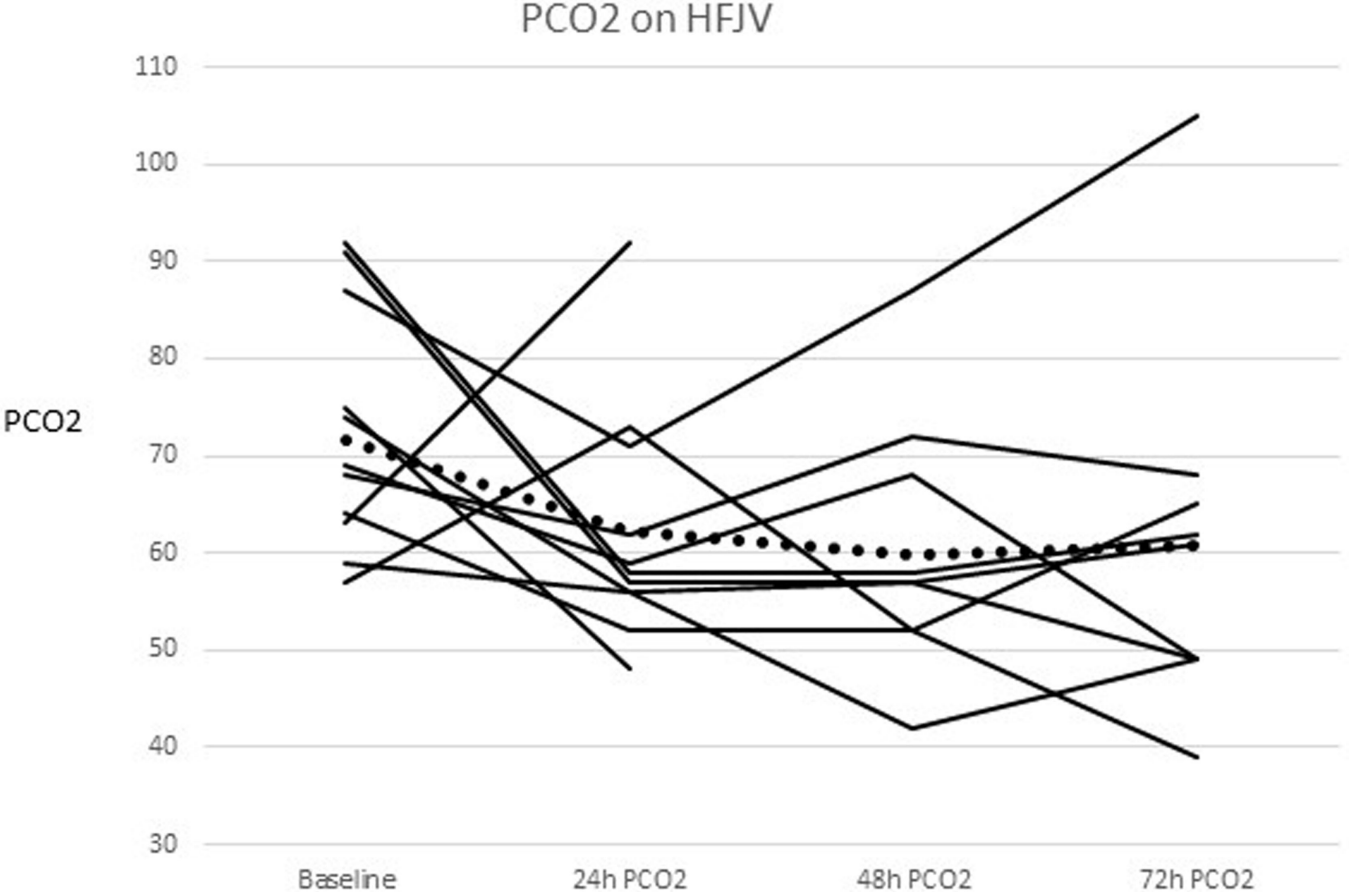
**PCO_2_ change for each individual and the combined mean after HFJV initiation**. The combined means are represented as the dotted line.

The mean PCO_2_ before HFJV initiation was 72 mmHg (range 57–102 mmHg), and decreased from baseline by 9 mmHg at 24 h, 12 mmHg at 48 h (*p* = 0.018), and 11 mmHg at 72 h after starting HFJV. Figure 1 shows individual patients as well as the mean PCO_2_ over time. The mean and median oxygenation index (OI) before starting HFJV were 17 and 14, respectively (range 5–32). The mean and median OI at 72 h were both 12 (range 2–24). There was no significant change in OI over this time frame.

None of the patients received ECMO. There were no complications of pneumothorax, pneumomediastinum, or subcutaneous emphysema associated with HFJV in this cohort. One patient developed subglottic stenosis. One patient died.

## Discussion

In patients with severe respiratory failure refractory to CMV, rescue therapies have been described, such as HFOV and ECMO (14–17). However, ECMO carries considerable morbidity and mortality. According to a query of the Extracorporeal Life Support (ELSO) registry from 1993 to 2007, the mortality of children who required ECMO for RSV pneumonia was 30%, and the mortality of children with chronic lung disease who required ECMO for respiratory failure was 41% (18). In addition to the risk of morbidity and mortality, an ECMO program requires substantial resources, expertise, and surgical support. Although a case report and small case series (16, 19, 20) describe the use of HFOV in obstructive airway disease, the use of HFOV in patients with severe obstruction to airflow has the potential to worsen air trapping and hyperinflation and may be considered a contraindication for use (21).

The pathophysiology of pediatric respiratory failure from RSV can be represented by a polarized clinical spectrum. One end of the spectrum is airway obstruction caused by bronchiolitis, which is characterized by airway edema, bronchial and bronchiolar obstruction, and mucus plugging. This can lead to wheezing, prolonged and incomplete exhalation with air trapping and auto- or intrinsic PEEP as well as radiographic evidence of hyperinflation. The other end of the spectrum of respiratory failure from RSV is pneumonitis characterized by pathophysiology similar to ARDS. This is manifested by edema and inflammation of the alveoli and pulmonary interstitium resulting in reduced compliance (7). The resulting atelectasis and multifocal airspace disease lead to intrapulmonary shunting, ventilation-perfusion mismatch, and hypoxemia. Although the clinical manifestations of RSV lower respiratory tract disease in any given patient can overlap, many patients exhibit a predominance of either obstructive or restrictive lung disease causing respiratory failure.

Maintenance of adequate FRC in patients with pneumonia/ARDS manifestations and avoidance of auto-PEEP and air-trapping in those with obstructive airways are major challenges. In some children, CMV fails to improve oxygenation/ventilation, and rescue therapies such as high frequency ventilation and ECMO are sought. If the outcomes were similar, HFV would be preferable to ECMO because of the less invasive nature and less technical support needed for implementation and management.

**Figure 2:**
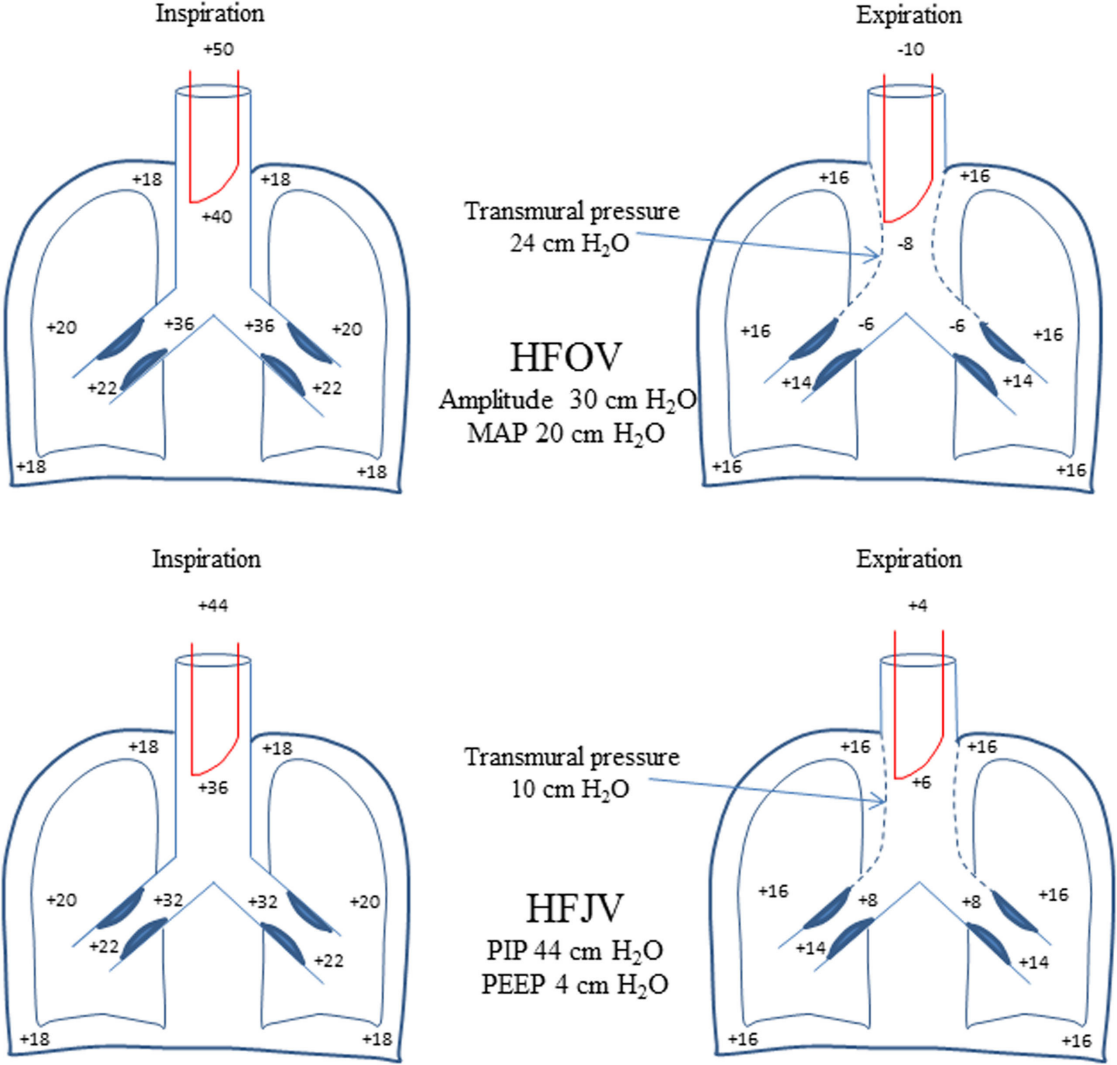
**Schematic comparison with theoretical pressures reflecting dynamic changes in airway caliber during HFOV and HFJV**. The active exhalation of HFOV with negative intraluminal pressure is expected to result in greater increase in transmural pressure compared with passive exhalation of HFJV where intraluminal pressure remains positive throughout exhalation. This would lead to greater collapse of the proximal airway and higher expiratory resistance and air trapping especially in an obstructive disease (such as RSV bronchiolitis) in HFOV compared with HFJV. Transmural pressure equals intrapleural pressure minus airway (alveolar) pressure. See text for further explanation.

In such patients, HFJV offers certain theoretical advantages compared to HFOV. The equal pressure point (EPP) where intrathoracic pressure during exhalation is equal to intraluminal pressure, is shifted distally (toward the alveoli) in diseases characterized by airway obstruction such as RSV bronchiolitis (22). The active expiration of HFOV creates negative pressure in the proximal airway that may not be uniformly dissipated distally because of prolonged time constant due to increased resistance. This leads to excessive transmural pressure resulting in proximal airway collapse, further increase in airway resistance, and air trapping. Transmural pressure favoring proximal airway collapse also occurs with the use of HFJV, but its magnitude is expected to be much less compared to that of HFOV because exhalation is passive and intraluminal pressure remains positive throughout exhalation (Figure 2).

High frequency jet ventilation offers some advantages in pulmonary hygiene over HFOV. Suctioning the airway may be done by way of an inline suction catheter with HFJV while the system is often opened for suctioning on HFOV, potentially resulting in alveolar derecruitment. Additionally, the physics of gas exchange peculiar to HFJV are beneficial in removing airway debris and in management of hyperinflation, both of which cause physiologic derangement in respiratory failure from RSV. HFJV delivers short pulses of high velocity inspired gas described as “transitional flow,” which preferentially travels much faster through the center of the airways where the drag is less. In RSV, these pulses of inspired air move past secretions coating the airways. The slower-moving exhaled gas spirals around the outer aspects of the airway, contributing to augmented removal of airway debris. This is consistent with previous reports describing the use of HFJV to augment the mobilization of obstructing casts in plastic bronchitis (23), and to remove debris in meconium aspiration (24).

The cohort of subjects in the present study represents the more severe spectrum of respiratory failure from RSV. Of 75 mechanically ventilated patients with acute respiratory failure from RSV infection, 11 were ill enough to be considered for rescue therapy. Nine of these 11 patients had pre-existing chronic lung disease of prematurity, which carries higher risk of mortality. Five patients had bronchopulmonary dysplasia, four of them being on supplemental oxygen at home. Three patients had a VI ≥ 70; one patient had an OI ≥ 32 prior to the initiation of HFJV. These patients represent the extremes of acute hypercapnic and hypoxic respiratory failure for whom extracorporeal support would be a reasonable option. They were successfully supported with HFJV until such time as they were able to be weaned off mechanical respiratory support. One patient developed subglottic stenosis, the other nine recovered to their pre-illness baseline for respiratory support. One patient in our study died, and that patient was deemed a poor candidate for ECMO due to pre-existing severe, chronic lung disease and prolonged cardiopulmonary arrest prior to arrival to the hospital.

Limitations of this study include the retrospective nature, small sample size, and lack of uniformity in HFJV management. However, the significance of these exploratory findings is the relative success of HFJV as an alternative treatment for severe ventilatory failure from RSV.

## Conclusion

This case series describes the utility of HFJV as an alternative, rescue therapy for RSV-induced respiratory failure that is refractory to CMV. HFJV was tolerated by this group of patients without evidence of new/worsened chronic lung disease. The utility of HFJV should be further explored in this patient population as an alternative therapy for severe, refractory ventilatory failure.

## Author Contributions

Dr. KV contributed to study design, data collection, data analysis, and the majority of the manuscript preparation. Dr. AAS contributed to study design, data analysis, preparation of the tables, and part of the manuscript preparation and revision. Dr. HS contributed to study design, data collection, and manuscript revision. Dr. APS contributed to study design, data analysis, preparation of the figures, and part of the manuscript preparation and revision.

## Conflict of Interest Statement

The authors declare that the research was conducted in the absence of any commercial or financial relationships that could be construed as a potential conflict of interest. The reviewer PK and handling Editor declared their shared affiliation, and the handling Editor states that the process nevertheless met the standards of a fair and objective review.
